# Connexin Mediated Cataract Prevention in Mice

**DOI:** 10.1371/journal.pone.0012624

**Published:** 2010-09-09

**Authors:** Lin Li, Catherine Cheng, Chun-hong Xia, Thomas W. White, Daniel A. Fletcher, Xiaohua Gong

**Affiliations:** 1 Vision Science Program and School of Optometry, University of California, Berkeley, California, United States of America; 2 Department of Physiology and Biophysics, State University of New York Stony Brook, Stony Brook, New York, United States of America; 3 Department of Bioengineering, University of California, Berkeley, California, United States of America; University of Florida, United States of America

## Abstract

Cataracts, named for any opacity in the ocular lens, remain the leading cause of vision loss in the world. Non-surgical methods for cataract prevention are still elusive. We have genetically tested whether enhanced lens gap junction communication, provided by increased *α*3 connexin (Cx46) proteins expressed from *α8(Kiα3)* knock-in alleles in *Gja8^tm1(Gja3)Tww^* mice, could prevent nuclear cataracts caused by the γB-crystallin S11R mutation in *Crygb^S11R/S11R^* mice. Remarkably, homozygous knock-in *α8(Kiα3/Kiα3)* mice fully prevented nuclear cataracts, while single knock-in *α8(Kiα3/−)* allele mice showed variable suppression of nuclear opacities in *Crygb^S11R/S11R^* mutant mice. Cataract prevention was correlated with the suppression of many pathological processes, including crystallin degradation and fiber cell degeneration, as well as preservation of normal calcium levels and stable actin filaments in the lens. This work demonstrates that enhanced intercellular gap junction communication can effectively prevent or delay nuclear cataract formation and suggests that small metabolites transported through gap junction channels protect the stability of crystallin proteins and the cytoskeletal structures in the lens core. Thus, the use of an array of small molecules to promote lens homeostasis may become a feasible non-surgical approach for nuclear cataract prevention in the future.

## Introduction

Cataracts, defined as any opacity in the lens, remain the leading cause of blindness in the world despite the success of surgical replacement with artificial lenses. A non-surgical method of cataract prevention remains an important and challenging topic. Age, mutated genes, radiation, smoking, chemical insults, physical injury and other systemic diseases can lead to cataract formation by initiating complicated pathological processes that result in abnormal protein aggregates and cellular disruptions in the lens [1], [2]. The bulk of the lens consists of precisely organized elongated fiber cells that are coupled by intercellular gap junction channels for maintaining lens homeostasis [3], [4], [5]. Interior lens mature fiber cells have minimum metabolism without any intracellular organelles and mainly contain crystallin proteins [2], [6]. Treatments using select small molecules, such as antioxidants, vitamins or ions, show conflicting results on the efficiency of cataract prevention in clinical trials [7], [8], [9], [10], [11]. This work has provided new evidence for the feasibility of cataract prevention via homeostasis regulation mediated by gap junction channels in animal models.

Crystallin proteins, classified as *α*, β and γ groups, are the main structural components of a mammalian lens [2]. Cataract formation is directly associated with the stability and solubility of lens crystallin proteins [2], [12], [13], [14]. Crystallin gene mutations are the most common cause of hereditary cataracts [15]. We have previously reported that γB-crystallin S11R mutation leads to a dominant congenital cataract, and homozygous *Crygb^S11R/S11R^* mutant mice develop severe nuclear cataracts regardless of the genetic background in the A/J, C57BL/6 and 129 strains [13]. Cataract formation was associated with abnormal degradation and aggregation of crystallins, disruption of membrane-cytoskeletal structures and the elevation of lens calcium level. Activated calcium-dependent proteases, such as calpains, are known to cleave crystallin proteins and to be associated with the degeneration of interior fiber cells in *Gja3^tm1^* knockout mice that lack intercellular gap junction communication consisting of *α*3 connexin (Cx46) proteins in the lens [16], [17], [18], [19].

At least three members of the connexin gene family, *α*1 connexin (Cx43), *α*3 connexin (Cx46) and *α*8 connexin (Cx50), encoded by *Gja1*, *Gja3* or *Gja8,* respectively, are utilized to form gap junction channels in the lens [5], [20]. Connexin 23, encoded by *Gjf1* and not verified to form gap junction channels, is also utilized in the embryonic lens [21], [22]. Gap junction channels consisting of *α*3 connexin and/or *α*8 connexin are essential for the lens transparency while only *α*8 connexin is important for the lens growth [19], [23], [24]. Interestingly, the role of *α*8 connexin in lens transparency can be compensated by the knock-in *α*3 connexin in *Gja8^tm1(Gja3)Tww^* mice [25]. Knock-in *α*3 connexins increase the cell-to-cell coupling between lens fiber cells [26]. We also reported that knock-in *α*3 connexin could suppress cataract formation caused by the *α*8 connexin G22R mutation in *Gja8^Lop10^* mice [27]. These studies indicate that knock-in *α*3 connexin enhanced intercellular gap junction communication may provide a potential way for promoting lens transparency and suppressing cataract formation by rescuing the deficiency of intercellular gap junction communication [25], [27], [28]. Here, we have investigated the molecular mechanism for how knock-in *α*3 connexin from *Gja8^tm1(Gja3)Tww^* mice suppressed the nuclear cataract caused by γB-crystallin S11R mutant proteins in *Crygb^S11R/S11R^* mice. In order to provide continuity from previous related functional studies of connexin and crystallin proteins, we have used simple names for labeling the data of different mutant mice - *α8(Kiα3/Kiα3)* for homozygous *α3* connexin knock-in (*Gja8^tm1(Gja3)Tww^/Gja8^tm1(Gja3)Tww^*), *α3(−/−)* for homozygous *α3* connexin knockout (*Gja3^tm1^/Gja3^tm1^*), *α8(−/−)* for homozygous *α8* connexin knockout (*Gja8^tm1^/Gja8^tm1^*) and *γB(S11R/S11R) for* homozygous *γ*B-crystallin S11R mutant (*Crygb^S11R/S11R^*).

## Results

### Prevention of nuclear cataracts

**Figure 1:**
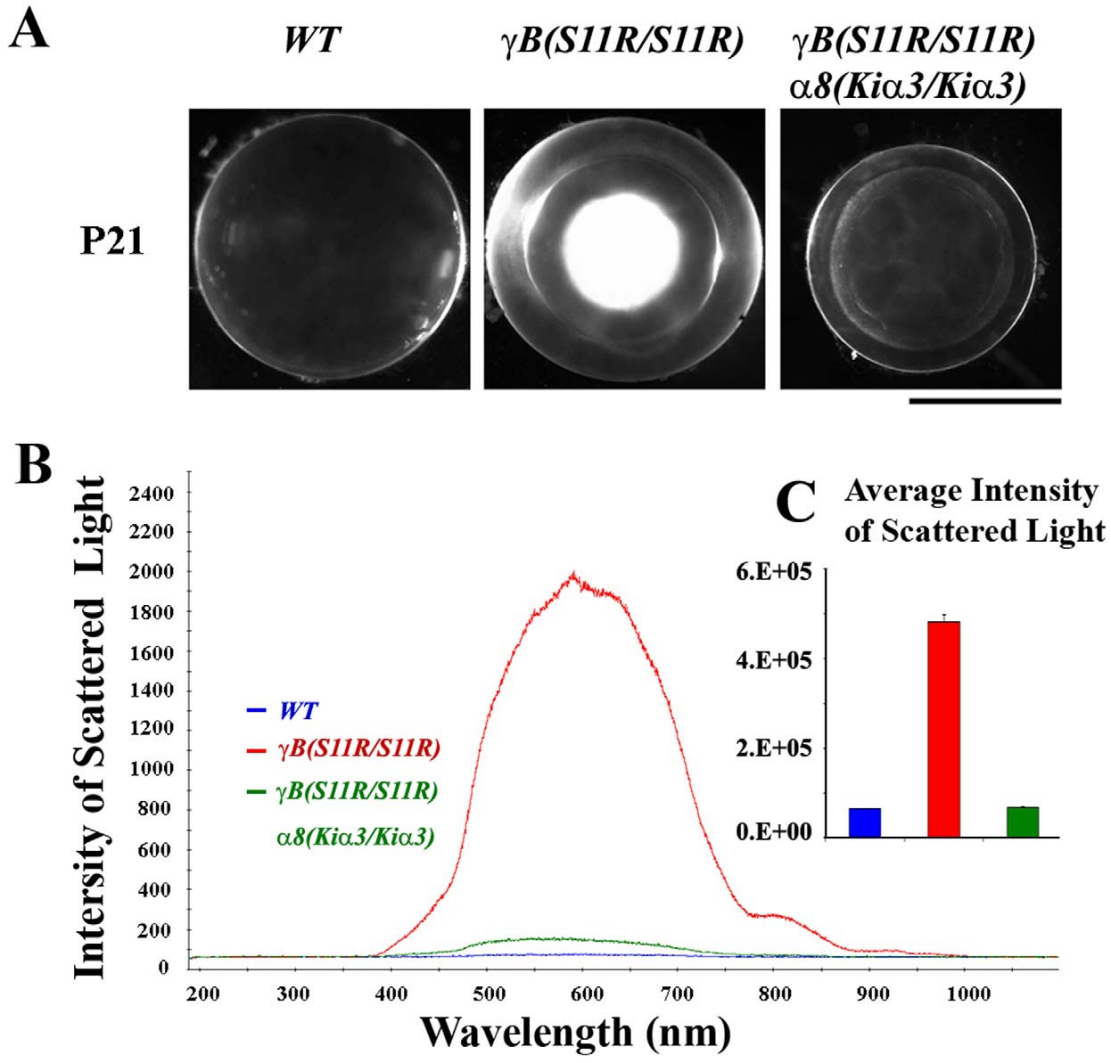
Cataract prevention mediated by *α*3 connexin in the *γ*B-S11R mutation. (A) Photos of wild-type (*WT*), mutant *γB(S11R/S11R)* and compound mutant *γB(S11R/S11R) α8(Kiα3/Kiα3)* lenses from P21 mice. Scale bar, 1 mm. (B) Representative light scattering graphs, obtained by an optical fiber and spectrometer, from P21 *WT* (blue), *γB(S11R/S11R)* (red) and *γB(S11R/S11R) α8(Kiα3/Kiα3)* (green) lenses. (C) The bar graph compares the normalized intensity of scattered light (y-axis with arbitrary units), calculated from the averaged area below the light scattering peaks of *WT*, *γB(S11R/S11R)* and *γB(S11R/S11R) α8(Kiα3/Kiα3)* lenses (x-axis).

To test whether increased *α*3 connexin proteins expressing from knock-in *α8(Kiα3/Kiα3)* alleles can prevent a severe nuclear cataract caused by the *γ*B-crystallin S11R mutant proteins expressing from *γB(S11R/S11R)* alleles, we generated compound mutant *γB(S11R/S11R) α8(Kiα3/Kiα3)* mice. Remarkably, the dense nuclear cataract was fully suppressed in compound mutant lenses while the very mild cortical opacity including an abnormal ring-like defect was remained (Figure 1A). The intensity of scattered light from these lenses was quantitatively measured using an optical fiber coupled with a spectrometer. Light scattering differences among wild-type, *γB(S11R/S11R)* and *γB(S11R/S11R) α8(Kiα3/Kiα3)* lenses were obvious (Figure 1B). Average intensity of scattered light (in arbitrary units) from 400 nm to 700 nm wavelength in wild-type (64434±749, N = 3), *γB(S11R/S11R)* (483415±17113, N = 4) and *γB(S11R/S11R) α8(Kiα3/Kiα3)* (68076±1852, N = 8) lenses was compared in a bar graph (Figure 1C). The basal level of the light intensity was defined as the level of wild-type (WT) lenses that do not scatter light. The intensity of scattered light from *γB(S11R/S11R) α8(Kiα3/Kiα3)* lenses had about 100-fold reduction in comparison to that from *γB(S11R/S11R)* lenses and had about a 6% increase in comparison to that from wild-type lenses. The compound mutant *γB(S11R/S11R) α8(Kiα3/Kiα3)* mice never developed dense nuclear cataracts up to one year of age. Thus, knock-in *α*3 connexin effectively prevents the dense nuclear cataracts caused by the γB-crystallin S11R mutation. Light scattering of the mild cortical cataract was insignificant.

### Preservation of lens interior fiber cells

**Figure 2:**
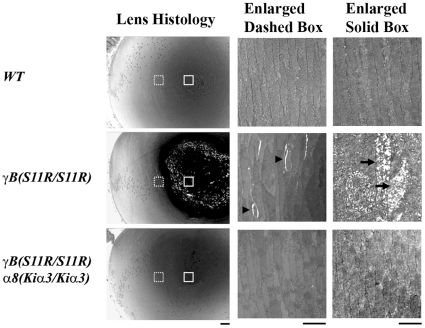
Histology of P7 *WT*, *γB(S11R/S11R)* and *γB(S11R/S11R) α8(Kiα3/Kiα3)* lenses. Wide field view of lens sections are shown on the left panels. Scale bar, 100 µm. High magnification views of selective interior fiber cells of lens sections are shown on the middle panels (dashed boxes, about 500–580 µm distance from the lens capsule) and the right panels (solid boxes, about 800–880 µm from the lens capsule). Uniformly elongated and tightly packed interior fiber cells are present in wild-type lens sections (the top panels) while irregularly elongated and loosely packed interior fiber cells (indicated by arrowheads on the middle panel) and disintegrated fiber cells (indicated by arrows on the middle-right panel) appears in *γB(S11R/S11R)* lenses. However, *γB(S11R/S11R) α8(Kiα3/Kiα3)* lens section displays uniformly elongated and tightly packed interior fiber cells without noticeable disintegrated fiber cells in the lens core (the bottom panels). Scale bars, 20 µm.

The dense nuclear cataract caused by the γB-crystallin S11R mutation was associated with the deterioration of intracellular components in fiber cells of the lens core (Figure 2). Enlarged extracellular spaces or disrupted fiber-to-fiber contacts were observed in deeper cortical fibers close to the degenerative mature fibers in the core of *γB(S11R/S11R)* lenses. In comparison, interior fiber cells of *γB(S11R/S11R) α8(Kiα3/Kiα3)* lenses appeared intact with appropriate cell organization without any histopathology observed in *γB(S11R/S11R)* lenses (Figure 2).

**Figure 3:**
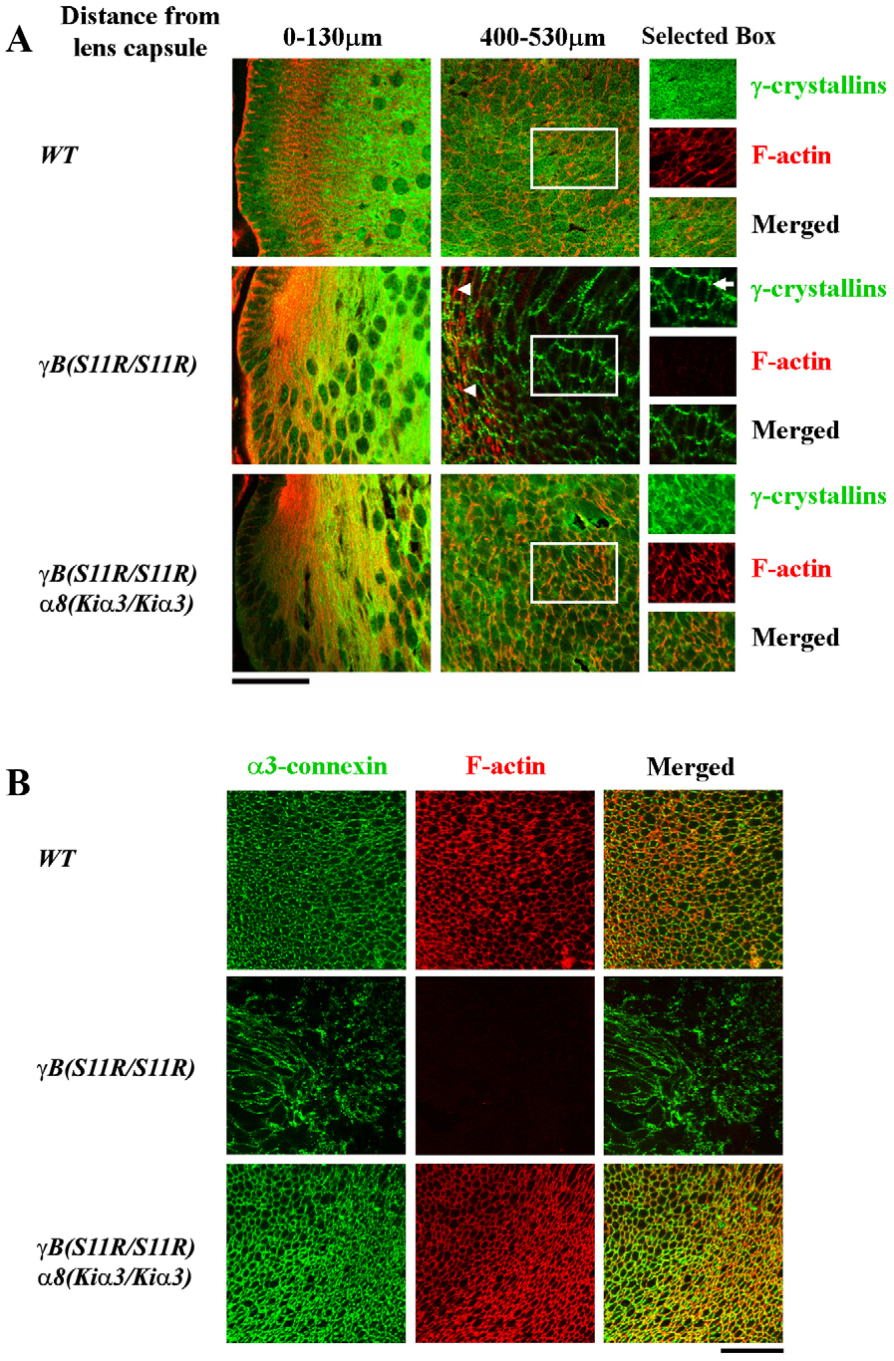
The distribution of actin filaments and cytosolic *γ*-crystallin in lens fiber cells. (A) Postnatal day 1 (P1) *WT*, *γB(S11R/S11R)* and *γB(S11R/S11R) α8(Kiα3/Kiα3)* lens frozen sections were stained with anti-*γ*-crystallin antibody (green) and rhodamine-phalloidin (red). Select areas (boxes, about 460–530 µm from lens capsule) reveal both separated and merged fluorescent images of *γ*-crystallin and F-actin in lens inner fiber cells. Wild-type lenses show typical F-actin staining and uniformly distributed *γ*-crystallins while *γB(S11R/S11R)* lenses show both aberrant F-actin (arrowheads) and *γ*-crystallin aggregates along cell-cell boundaries (without F-actin, an arrow) in lens inner regions. Remarkably, *γB(S11R/S11R) α8(Kiα3/Kiα3)* lenses display normal F-actin with cytosolic *γ*-crystallins in inner fiber cells. Scale bars, 50 µm. (B) Enlarged view and merged fluorescent images of immunostained *α*3-connexin (green) with rhodamine-phalloidin-stained F-actin (red) in inner fiber cells of *WT*, *γB(S11R/S11R)* and *γB(S11R/S11R) α8(Kiα3/Kiα3)* lenses from P7 mice. Scale bars, 50 µm.

Immunohistological staining results of F-actin and γ-crystallin revealed that *γB(S11R/S11R)* lenses had substantial membrane-associated γ-crystallin aggregates, a lack of F-actin and extremely low levels of cytosolic γ-crystallin in interior fiber cells. But *γB(S11R/S11R) α8(Kiα3/Kiα3)* lenses showed normal distribution of F-actin and cyotosolic γ-crystallin in interior fiber cells, similar to that of wild-type lenses (Figure 3A). Punctuate *α*3 connexin staining was present at the plasma membranes of wild-type lens fiber cells (Figure 3B). Although punctuate *α*3 connexin signals still appeared in altered fiber cells of *γB(S11R/S11R)* lenses, much stronger *α*3 signals were present in *γB(S11R/S11R) α8(Kiα3/Kiα3)* lens fiber cells (Figure 3B). F-actin staining was absent in fiber cells from the *γB(S11R/S11R)* lens core, but F-actin signals appeared normal in *γB(S11R/S11R) α8(Kiα3/Kiα3)* lenses at the same age. Thus, knock-in *α*3 connexin protected the integrity of lens interior fiber cells probably by maintaining normal distribution and properties of F-actin and γ-crystallin proteins.

### Inhibition of calcium elevation and crystallin degradation

**Figure 4:**
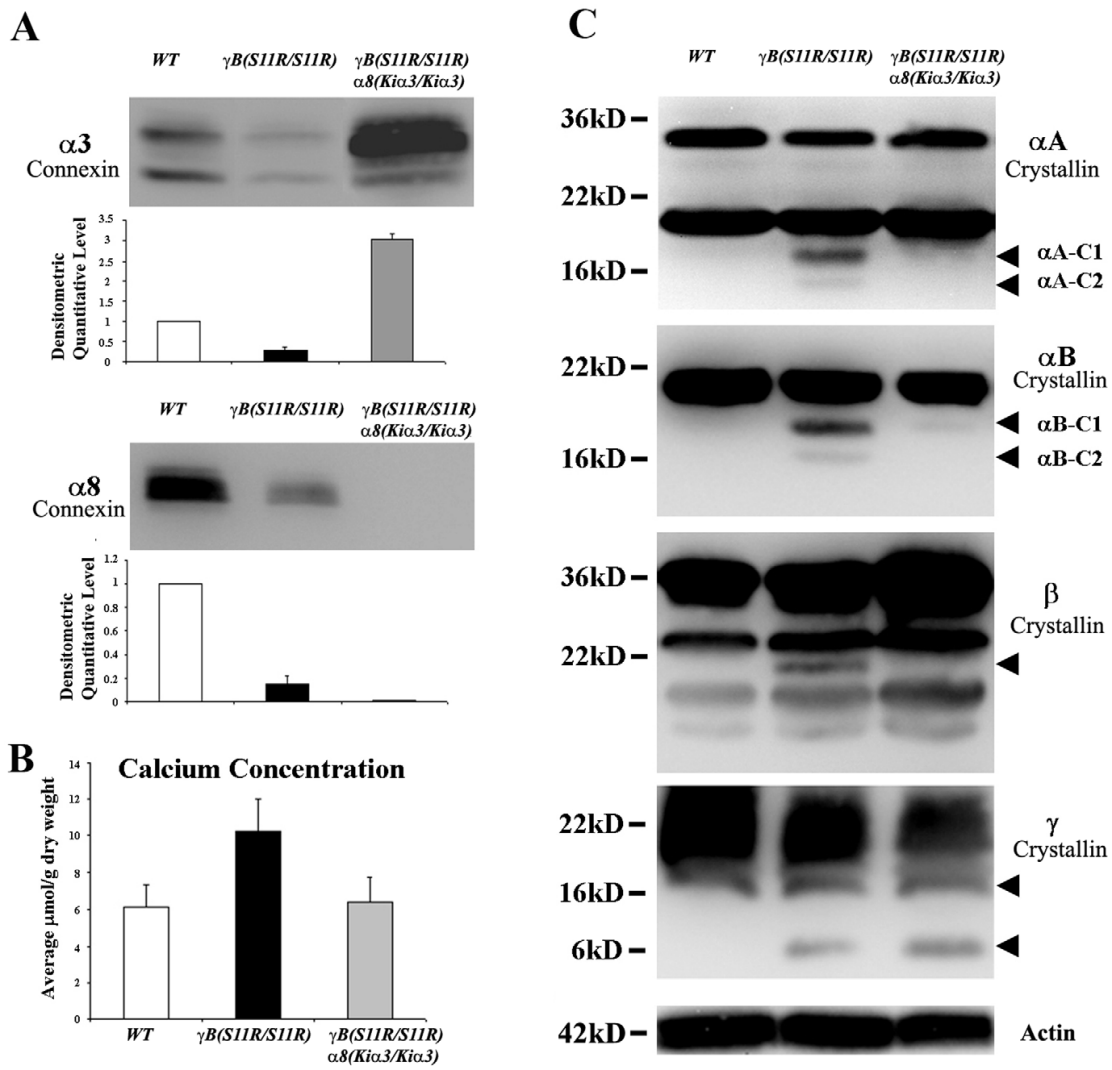
Levels of lens connexins and total lens calcium. (A) Western blotting images and densitometric quantifications of the expression level of *α*3 connexin and *α*8 connexin in P10 *WT*, *γB(S11R/S11R)* and *γB(S11R/S11R) α8(Kiα3/Kiα3)* lenses. (B) Total lens calcium levels in P10 *WT*, *γB(S11R/S11R)* and *γB(S11R/S11R) α8(Kiα3/Kiα3)* lenses. (C) Western blotting images of lens total *α*A-, *α*B-, β-, *γ*-crystallin and actin. Arrowheads indicate the cleaved forms of crystallins. Two major cleaved forms of *α*A- and *α*B-crystallins are marked as *α*A-C1, *α*A-C2, *α*B-C1 and *α*B-C2, respectively.

It is known that the *α8(Kiα3)* allele increases the level of *α*3 connexin proteins and elevates the electrical coupling between interior mature fiber cells [26]. Alpha8 connexin was not expressed in *γB(S11R/S11R) α8(Kiα3/Kiα3)* lenses because the *α8(Kiα3)* allele replaced the endogenous wild-type *α8* connexin gene. Densitometric quantification of Western blotting results show that *α*3 connexin protein levels decreased about 4-fold in P10 *γB(S11R/S11R)* lenses and increased about 3-fold in P10 *γB(S11R/S11R) α8(Kiα3/Kiα3)* lenses, compared to that in postnatal day 10 (P10) wild-type lenses (N = 3, P<0.05) (Figure 4A). In addition, about a 5-fold reduction of *α*8 connexin levels was detected in the γB(S11R/S11R) lenses (N = 3, P<0.05) (Figure 4A). However, neither endogenous *α*3 nor *α*8 connexin proteins were reduced in P1 *γB(S11R/S11R)* mutant lenses (data not shown).

Lens total calcium level was measured by an inductively coupled plasma-optical emission spectrometry (ICP-OES). In comparison to wild-type lenses, *γB(S11R/S11R)* lenses had substantially increased total calcium level while the calcium level in *γB(S11R/S11R) α8(Kiα3/Kiα3)* lenses remained unchanged (Figure 4B). All the differences were statistically significant (N = 5, P<0.004). Moreover, we examined calcium-dependent crystallin protein degradation and found that cleaved *α*A-, *α*B-, β- and γ-crystallins were present in *γB(S11R/S11R)* lenses but were almost absent in *γB(S11R/S11R) α8(Kiα3/Kiα3)* lenses with a very small amount of cleaved *α*B-crystallin (*α*B-C1) and γ-crystallin (Figure 4C). These results suggest that the elevated level of *α*3 connexin suppressed calcium elevation to prevent the degradation of *α*- and β-crystallins and nuclear cataract formation in *γB(S11R/S11R) α8(Kiα3/Kiα3)* lenses.

### Dosage effect of *α*3 connexin on cataract prevention

We further evaluated whether endogenous *α3* connexin genes were required for the cataract prevention mediated by *α8(Kiα3)* alleles and whether one copy of the *α8(Kiα3)* allele was sufficient for cataract prevention. Triple mutant mice, *γB(S11R/S11R) α8(Kiα3/Kiα3) α3(−/−)* and *γB(S11R/S11R) α8(Kiα3/−) α3(−/−)*,were generated by breeding *α3(−/−)* knockout mice with the compound mutant *γB(S11R/S11R) α8(Kiα3/Kiα3)* mice. The first triple mutant *γB(S11R/S11R) α8(Kiα3/Kiα3) α3(−/−)* mice, without endogenous *α*3 connexin and with two copies of the *α8(Kiα3)* allele, developed nuclear cataracts between 4 months to 1 year of age (data not shown). Thus, endogenous *α*3 gene is needed for cataract prevention in aged mice.

**Figure 5:**
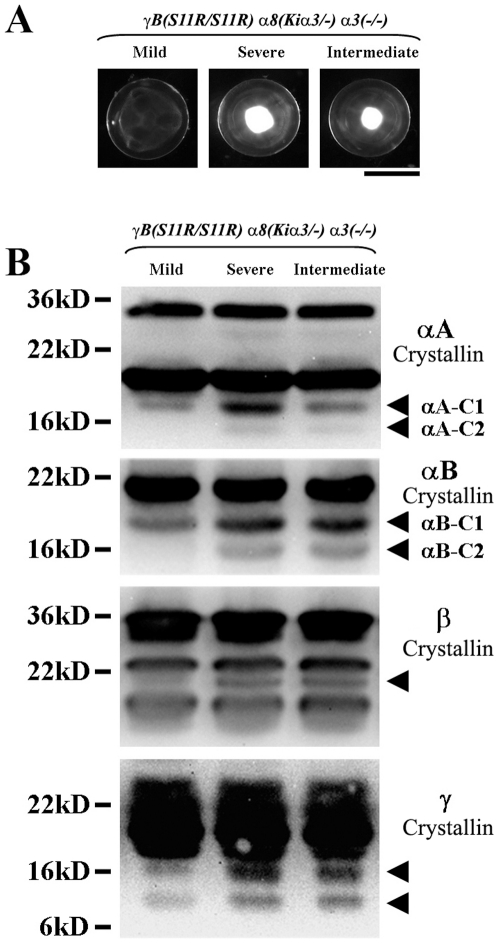
Correlation between cleaved crystallin proteins and the severity of nuclear cataracts. (A) Lens photos from three *γB(S11R/S11R*) *α8(Kiα3/−) α3(−/−)* littermates at the age of one month. Scale bar, 1 mm. (B) Western blotting images of lens total *α*A-, *α*B-, β- and *γ*-crystallins. Arrowheads indicate the cleaved forms of crystallins. Two major cleaved forms of *α*A- and *α*B-crystallins are marked as *α*A-C1, *α*A-C2, *α*B-C1, and *α*B-C2, respectively.

The second triple mutant *γB(S11R/S11R) α8(Kiα3/−) α3(−/−)* mice, with one copy of the *α8(Kiα3)* allele and without endogenous wild-type *α3* connexin, displayed variable lens phenotypes. Littermates displayed clear lenses or cataractous lenses with mild, intermediate or dense nuclear opacities at the age of one month (Figure 5A). Occasionally, the left and right lenses of the same mouse displayed varying degrees of nuclear opacity (data not shown). Western blotting results showed that increases of cleaved forms of *α*A-crystallin (*α*A-C1 and -C2), *α*B-crystallin (*α*B-C1 and -C2) and β-crystallin were correlated with the severity of nuclear cataracts (Figure 5B). The levels of cleaved crystallin (*α*A-C1) appeared to be closely correlated to the severity of nuclear cataracts in *γB(S11R/S11R) α8(Kiα3/−) α3(−/−)* lenses. These results indicate that one copy of *α8(Kiα3)* could only partly inhibit or delay the development of nuclear cataracts. Thus, the dosage of *α*3 connexin proteins expressed from knock-in *α8(Kiα3)* alleles and endogenous *α3* connexin gene alleles was important for prevention or suppression of nuclear cataracts.

## Discussion

We have demonstrated that elevated *α*3 connexin expression can prevent hereditary nuclear cataracts caused by the γB-crystallin S11R mutation. Morphological evidence indicates that the prevention of this nuclear cataract is correlated with the inhibition of inner fiber cell degeneration and the maintenance of membrane-cytoskeleton structures and crystallin stability and/or solubility. Presumably, enhanced exchange of metabolites or ions transported by gap junction channels composed by *α*3 connexin is responsible for preventing calcium level elevation, crystallin aggregation and degradation in the lens. Therefore, this work proves the principle that cataract prevention can be achieved by promoting intercellular gap junction communication in the lens.

Cataracts are ultimately associated with aggregation and/or degradation of crystallin proteins regardless of the primary causes, such as age, genetic disorders or radiation damage. Nuclear cataracts are associated with disturbed membrane-cytoskeletal structures as well as with crystallin protein degradation and/or modifications in the lens core. Our work supports the notion that nuclear cataract prevention may require an array of metabolites or reagents, transported by gap junction channels, to prevent aberrant changes in lens proteins and to maintain lens homeostasis needed for lens transparency [29]. Calcium homeostasis is known to play important roles in the regulation of lens transparency [16], [17], [18]. The two main mammalian calpains, 1 and 2, are heterodimers of a large 80 kDa subunit and a small 28 kDa subunit that together bind multiple calcium ions during enzyme activation. Calpains seem to be important in the regulation of lens transparency and cataract prevention [18], [30], [31]. The activation of calpains in mouse lens is known to be related to the cleavages of *α*A- and *α*B-crystallins [32]. Presumably, the level of cleaved forms of *α*A- and *α*B-crystallins was dependent on the degree of calpain activation in the lens. Mutant γB-crystallin S11R proteins induce abnormal protein aggregation that probably disrupts membrane-cytoskeleton structures of inner fiber cells. Subsequently, increased calcium influx and activation of calcium-dependent protein degradation lead to the degeneration of inner mature fiber cells and a dense nuclear cataract. Probably by enhancing cell-cell communication, knock-in *α*3 connexin prevents calcium level elevation and the activation of calpains, which in turn prevents the degradation of *α*- and β-crystallins in the lens. The cleavage of γ-crystallin in connexin *α3(−/−)* knockout mice is related to the activation of calpain 3 (or Lp82 protease) [17]. However, the protease that is involved in the cleavage of γ-crystallin in *Crygb^S11R/S11R^* mutant mice remains unknown.

This work demonstrates that it is feasible to prevent nuclear cataract formation by directly promoting lens homeostasis via increased gap junction communication. The mechanism for how *α*3 connexin leads to a dosage-dependent inhibition of nuclear cataract induced by γB-crystallin S11R mutation needs to be further investigated. It is very interesting that the presence of a low level of *α*3 connexin, expressed from one copy of the knock-in *α8(Kiα3)* allele, shows incomplete suppression of this nuclear cataract. This result supports the observations of previous studies that other genetic modifiers play a significant role in the severity of nuclear cataracts when gap junction communication is insufficient in the lens and that the heterogeneity of nuclear cataracts was associated with the 129 and C57B6 mouse strain backgrounds [33], [34]. Thus, identification of any of these genetic modifiers will be valuable for understanding the mechanisms of connexin-mediated cataract formation and prevention.

It is possible that the level or the amount of small metabolites or ions that pass through gap junction channels composed of *α*3 connexin is the key to understand *α*3 connexin-mediated prevention of nuclear cataracts. Although it is difficult to increase the numbers of gap junction channels in any mature lenses, it may be practical and effective to supplement key metabolites and ions transported through existing gap junctions to enhance lens homeostasis to prevent or delay age-related cataracts. This notion is supported by recent studies that vigorous physical activities, which elevate systemic metabolism, reduced the risk for age-related cataracts as well as age-related macular degeneration [35]. Thus, the identification of an array of small molecules, transported by gap junction channels and needed for lens transparency, will be the next important step not only for understanding the lens homeostasis but also for developing a non-surgical method for cataract prevention [8].

## Materials and Methods

### Generation and genotyping of compound and triple mutant mice

Mouse care and breeding were performed according to an animal protocol (protocol#: R280-1210) approved by the Animal Care and Use Committee at University of California, Berkeley and the ARVO Statement for the Use of Animals in Ophthalmic and Vision Research. Mouse pupils were dilated with 1% atropine and 1% phenylephrine before the eyes were examined for lens clarity by a slit lamp.

Homozygous *Crygb^S11R/S11R^* (or *γB(S11R/S11R)*) mice were crossed with homozygous knock-in *Gja8^tm1(Gja3)Tww^/Gja8^tm1(Gja3)Tww^* (or *α8(Kiα3/Kiα3)*) mice to generate double heterozygous compound mutant *γB(S11R/+) α8(Kiα3/+)*, which were then mated with *Crygb^S11R/S11R^* (or *γB(S11R/S11R)*) mice to generate *γB(S11R/S11R) α8(Kiα3/+)* mice. Homozygous compound mutant *γB(S11R/S11R) α8(Kiα3/Kiα3)* were generated from the intercross of *γB(S11R/S11R) α8(Kiα3/+)* mice. The triple mutant mice, *γB(S11R/S11R) α8(Kiα3/Kiα3) α3(−/−)* or *γB(S11R/S11R) α8(Kiα3/−) α3(−/−)*, were generated through several mating steps between *γB(S11R/S11R) α8(Kiα3/Kiα3)* mice and *α3(−/−)* (or *Gja3^tm1^/Gja3^tm1^*) knockout or *α8(−/−)α3(−/−)* (or *Gja8^tm1^/Gja8^tm1^ Gja3^tm1^/Gja3^tm1^*) double knockout mice. *Crygb^S11R/S11R^* mutant mice had wild-type *Bfsp2* (or *CP49*) through 4 generations of backcrossing with the C57BL/6J strain. We confirmed that previously reported mutant lines including *Gja8^tm1(Gja3)Tww^/Gja8^tm1(Gja3)Tww^*, *Gja3^tm1^/Gja3^tm1^* and *Gja8^tm1^/Gja8^tm1^ Gja3^tm1^/Gja3^tm1^*, were maintained in C57BL/6J strain background and contained wild-type *Bfsp2* (or *CP49*). Based on an arbitrary prediction, all compound mutant mice had no more than 93.75% genetic background of the C57BL/6J strain and at least 6.25% genetic background of the AJ strain. Since both *Gja3* (*α*3 connexin) and *Gja8* (*α*8 connexin) knockout mice were originally generated from J1 ES cells of the 129SvJae mouse strain, these mutant mice probably had 129SvJae alleles on chromosomes 14 and 3 where *Gja3^tm1^* and *Gja8^tm1^* are located, respectively.

The *Crygb^S11R^* mutant and wild-type *Crygb* alleles can be genotyped by using PCR with satellite marker D1Mit156 (left: TCTGCTGCCACTTCTGAGAA; right: TGTGTGTCTATGGACATGGATG). The *Crygb^S11R^* mutant allele originated from the AJ strain background displayed an 112 bp PCR fragment while the wild-type *Crygb* allele from the C57BL/6J strain background yielded a 143 bp PCR fragment. The genotyping of *Gja3^tm1^* and *Gja8^tm1^* mutant alleles was assessed by PCR as described previously [19], [24].

### Fresh lens imaging and light scattering quantification

Fresh lenses, dissected from enucleated eyeballs, were immediately immersed in PBS at 37°C and imaged under a Leica MZ16 dissecting scope using a digital camera. The intensity of light scattered by lenses was measured using the HR 2000CG_UV_NIR High Resolution Spectrometer and a QP400-2-UV-VIS fiber optic cable (Ocean Optics, Dunedin, FL, USA). Lenses were illuminated by a white light source perpendicular to the equator of the lens. Intensity of scattered light was captured by the optical fiber with a whole acceptance of angle of 24.8°. Spectrums were recorded and saved for later comparison. Each lens was measured twice in succession to show repeatability. The intensity of the illuminating light was kept constant between measurements. The measurements were represented as graphs with wavelength on the x-axis and intensity of scattered light on the y-axis. Data were stored as ASCII files, and the area under the curve was calculated by a Matlab program. Student's t-test was used for statistical analysis. P values <0.001 were considered significant.

### Histology

Enucleated eyeballs opened at the anterior chamber or posterior vitreous were immersed in a fixative solution containing 2% glutaraldehyde and 2.5% formaldehyde in 0.1 M cacodylate buffer (pH 7.2) at room temperature for 5 days. Samples were postfixed in 1% aqueous OsO4 and then dehydrated through graded acetone. Samples were embedded in eponate 12-araldyte 502 resin (Ted Pella, Redding, CA, USA). Lens sections (1 µm thick) across the equatorial plane were collected on glass slides and stained with toluidine-blue. Bright-field images were acquired using a light microscope (Axiovert 200; Carl Zeiss, Oberkochen, Germany) with a digital camera.

### Immunohistochemistry and confocal microscopy

Mouse eyes were fixed with fresh 4% formaldehyde in phosphate-buffered saline (PBS) for 30 minutes, then washed with PBS and soaked overnight in 30% sucrose in PBS. Afterward, the samples were processed and sectioned with a Cryostat 1900 (Leica, Germany) using a standard frozen-section method [19]. Tissue sections were washed with three exchanges of sterile PBS (10 minutes each time), followed by blocking (3% BSA, 3% NGS, 0.01% Triton X-100 in PBS) for 1 h at room temperature. Antigens were labeled using primary antibodies (see detail information in western blot analysis) for 1 hour at room temperature (or overnight at 4°C). After three more washes with sterile PBS, samples were incubated with secondary antibodies (Invitrogen, Carlsbad, CA, USA) for 1 hour at room temperature. Slides were then mounted with Vectashield Mounting Medium with DAPI (Vector Laboratories, Burlingame, CA, USA) after washing in PBS. The distribution of antigens was analyzed by a laser confocal microscope (Leica, Wetzlar, Germany).

### Western blot analysis

Enucleated fresh lenses were weighed and homogenized directly in the sample buffer (60 mM Tris, pH 6.8, 2% SDS, 10% glycerol, 5% β-mercaptoethanol, and 0.001% bromophenol blue) to prepare lens total proteins. To prepare the NaOH-insoluble protein samples, two lenses were homogenized in 0.1 M NaCl and 50 mM Na_2_HPO_4_ (pH 7) at the ratio of 40 mg lens wet weight/ml solution. The insoluble material was collected after centrifugation at 15,000 rpm for 15 min and washed once with the same solution. The insoluble pellet was further homogenized in 0.5 ml of 20 mM NaOH and 1 mM NaHCO_3_ solution. Again, an insoluble pellet was collected after centrifugation at 15,000 rpm for 15 min and washed once with 20 mM NaOH and 1 mM NaHCO_3_ solution. This insoluble pellet was dissolved in sample buffer. Equal volumes of samples were loaded on a 12.5% SDS-PAGE gel for separation, and separated proteins were transferred to a polyvinylidene difluoride membrane (Bio-Rad, Hercules, CA, USA). Lens crystallin and connexin proteins were detected by Western blotting with rabbit polyclonal antibodies against *α*- and γ-crystallins (generously provided by Dr. Joseph Horwitz at the University of California, Los Angeles), β-crystallin (generously provided by Dr. J. Samuel Zigler at the National Eye Institute), *α*3 connexin (generated previously in the lab), *α*8 connexin (generously provided by Dr. J. Mario Wolosin Mount Sinai School of Medicine, New York) and a mouse monoclonal antibody against β-actin (Sigma, St. Louis, MO, USA). More than three sets of lens protein samples from different mice were examined, and representative data were shown.

### Ion concentration measurement

Lens ion concentration was measured by inductively coupled plasma–optical emission spectrometry (ICP-OES) from a core facility at the University of California, San Diego. The method was described previously [18]. Twenty lenses were dissected from each mouse line and then immediately subjected to vacuum drying for 48 hours. Dry lenses were weighed and solubilized in 500 µL nitric acid (33.5∼35%; Fisher Scientific, Pittsburgh, PA, USA) for 12 hours at 37°C with shaking and then diluted with water into 3 ml. Samples were further diluted to reach ion concentrations of 20 ppb to 1 ppm for measurement. According to the estimated sample ion concentration, a series of standards were made. Ion concentrations were determined by their intensities acquired by the instrument. Measurement error was approximately 3%. Ion concentrations were normalized by lens dry weight.
